# Trends in the use of the Internet for health purposes in Poland

**DOI:** 10.1186/s12889-015-1473-3

**Published:** 2015-02-27

**Authors:** Maria Magdalena Bujnowska-Fedak

**Affiliations:** Department of Family Medicine, Wroclaw Medical University, Syrokomli 1, 51-141 Wrocław, Poland

**Keywords:** Internet use, Internet use for health purposes, Trends, Patterns, Sources of health information, Profile of Internet user, Cross-sectional

## Abstract

**Background:**

In Poland, like in other European countries and in accordance with the global trend, the number of computer users and people who have access to the Internet has increased considerably. The study investigates trends and patterns of Polish health-related Internet use over a period of seven years. The main objective of the study was to estimate the change in the proportion of the population using Internet for health purposes and to show the potential trend in perceptions and preferences of Polish citizens in this respect as well as factors affecting their use.

**Methods:**

The study was based on three national surveys that were conducted in 2005, 2007, and 2012. A total of 3027 adult citizens were selected randomly from the Polish population. A sample collection was carried out by Polish opinion poll agencies by computer-assisted telephone interviews. The subjects were asked to respond to general questions about their Internet use and their Internet use for health-related purposes, as well as to express their opinions about various sources of medical information, frequency, and the need for direct communication with health professionals via the Internet and other interactive forms of online activities.

**Results:**

The proportion of the Polish population that used the Internet for health-related purposes increased significantly (41.7% in 2005, 53.3% in 2007, and 66.7% in 2012). The Internet has become an important source of health information for almost half of Polish citizens, overtaking television, radio, press, and courses or lectures in the ranking list. As the medium matures, the use of interactive, health-related online services has also increased remarkably. However, while the main users of the Internet are certainly younger people, the largest growth potential has been observed among the elderly. The profile of the most likely Internet user and the citizen for whom the Internet is an important source of health information has been determined.

**Conclusions:**

The Internet offers enormous opportunities, particularly for providing and improving consumer information services with regard to health care. A sharply increasing trend regarding Internet use, Internet use for health purposes, and the interactive use of the Internet related to health has been observed among Polish citizens.

**Electronic supplementary material:**

The online version of this article (doi:10.1186/s12889-015-1473-3) contains supplementary material, which is available to authorized users.

## Background

E-health, defined as the use of emerging information and communication technology (ICT) and especially the Internet for health related matters, has become an important supplement to traditional health resources [1,2]. E-health holds the potential to improve health care access, support information exchange, reduce costs, and improve the quality of patient care [1,3,4]. New means of communication (e-health technologies, ICT) can offer innovations that improve the public and individual health through personalized medicine and aggregated health data [5].

The deployment of e-health services at the point of care is to a large extent consumer-driven. The concept of the “e-health consumer” includes patients, patients’ friends and relatives, and citizens in general who use the Internet and innovative ICT technologies to make informed decisions about their health [6]. The Internet offers enormous opportunities, particularly for providing and improving consumer information services with regard to health care. Interactivity and the large amount of information available via the Internet empower citizens to take better care of themselves and to communicate with their doctors. The Internet allows providers to deliver citizen-centered care faster and more efficiently [1,4,7].

The Internet is a phenomenon that no one could have predicted still in the beginning of 1990s. According to International Telecommunication Union (ITU), approximately 1 in 4 people in the world are now online, including nearly 3 in 4 citizens of the developed world [8]. In Poland, which is consistent with European and global trends, the number of computer users and people who have access to the Internet has increased considerably over the past decade [9-15]. According to Berezowska et al. [16], 54% of Polish households were equipped with a computer in 2007 and 41% had access to the Internet. In 2012, 73% of households possessed computers and approximately 71% had Internet access [17]. The Internet has been regularly used by 58% of Polish adult population, and more than 57% of Internet users (IU) have found their ability to use computers and the Internet sufficient to communicate using online tools [16]. Among various objectives found for Internet use, using it for health purposes occupies a prominent place. Similar to residents in other countries, Polish citizens are increasingly exposed to health services offered by the Internet [1,9-11,13-15,18-25].

Certainly, the main use of the Internet for health-related matters is to seek information, as the medium is an inexhaustible source of a variety of health information [9,10,13,15,19,23,26-28]. Other activities, such as interacting with health professionals, accessing and managing one’s own personal health records, participating in forums or self-help groups focusing on health and illness, or ordering medicine and other medicinal products, are less frequently conducted by consumers. Their prevalence, however, is growing as the medium matures and IU become more experienced [11,18,22,29-31].

This study complements preceding surveys in a particularly interesting way by investigating trends and patterns of Polish health-related Internet use over the last seven years. The main objective of the study is to estimate the change in the proportion of the population using the Internet for health purposes and to show the potential trend in perceptions and preferences of Polish citizens regarding Internet use as well as factors affecting their usage. Moreover, to shed some light on the future, the study draws the most likely profile of IU among the Polish population in general and in the subgroups of those who consider the Internet an important source of medical information.

## Methods

### Participants and procedure

The study was based on three national surveys conducted in 2005, 2007, and 2012. The first two surveys were conducted within the scope of the WHO/European e-Health Consumer Trends project, and the study is a secondary analysis of the data set. Primary analysis of the collected data was published by Bujnowska et al. and Staniszewski et al. [14,15,32]. Some of the data were also used in other papers related to WHO project [10,11,30,31]. The third survey, conducted in 2012, is a separate population-based study that has never been published before. The paper is based on the primary analysis of the collected data.

During three surveys, a total of 3027 respondents (1027 in 2005, 1000 in 2007, and 1000 in 2012) were selected from among the Polish population. Data were obtained from a sample of adults in all age categories (aged 15–80+ years). Sample collection was carried out by Polish opinion poll agencies (CBOS, TNS) through computer-assisted telephone interviews (CATI) in November 2005, April 2007, and October-November 2012. Random digital dialing in strata was used to ensure a randomized representative sample of the population. Quotas were constructed based on census data for age, gender, and place of residence (size of the place of residence and region of the country) to make sure the data was representative in this regard. Sampling continued until at least 1,000 complete interviews were collected. Both landline telephones and mobile phones were included in the survey. The polling agencies conducting the interviews were instructed to follow standard procedures related to a contact with a replacement if a person originally selected for interview was unavailable. No variables had more than 5% of missing data.

When this sampling procedure is used, calculating a response rate is challenging because a required number of responses is set before sampling starts. The number of calls reaching the target person can be divided into two groups: “no contact”, including incorrect numbers, not answering the phone, disconnected numbers, answering machines, and “non-responses,” including people not wanting or not having time to participate in the interview, too sick to take part, with language problems, and interrupted interviews. If we want to get a response rate for telephone interviews comparable to ordinary interviews, it seems reasonable to exclude the “no contact group” and to calculate a response rate among persons who in fact had a chance to participate. In 2005, one could not get a response rate because “non-responses” and “non-contacts” were not properly recorded. In 2007 and 2012, using “non-responses” group for calculation, an average response rate was 33.5% (32.8% in 2007 and 34.2% in 2012).

### Measures

The questionnaire used in the study (as with all surveys) was designed for CATI. The main questions used in the analysis are shown below. Initially, the subjects were asked to respond to questions about general Internet use and the Internet use for health-related purposes. General Internet use was measured with the question: “How often do you use the Internet?” The response categories were “Every day,” “Every week,” “Every month,” “Less than once a month,” “I have never used the Internet,” and “I have never used it, but I have asked others to do it for me.” The initial six options were grouped into two categories: Internet Users (“Every day,” “Every week,” “Every month,” and “Less than once a month”) and Internet Non-Users (“I have never used the Internet” and “I have never used it, but I have asked others to do it for me”). Internet use for health-related purposes was measured with the question: “How often do you use the Internet to get information about health or illness?” The response alternatives were: “Every day,” “Every week,” “Every month,” “Every six months,” “Every year,” “Less than once a year,” and “Never.” Those not answering “Never” were coded as Internet Users for Health Purposes. The importance of various health information channels was assessed with the statement: “I will now read a list of various sources of information about health or illness, and would like to know how important these are to you.” The responses to this item were provided according to the five-point Likert scale from “Not important” to “Very important” with the neutral response in the middle position. Respondents were subsequently invited to express their opinions about different patterns for using the Internet for health purposes and the frequency of their use. The need for searching the Internet to get information about health or illness was measured with the question: “How often do you use the Internet to read about health and illness?” The response categories were “Every day,” “Every week,” “Every month,” “Every six months,” “Every year,” “Less than once a year,” and “Never.” They were grouped into categories “At least once a year” (“Every day,” “Every week,” “Every month,” “Every six months,” and “Every year”), “Less than once a year,” and “Never” for better clarity of analysis and the possibility to compare results with similar European surveys [11]. Direct communication with health professionals (both known and unknown) via the Internet and other interactive forms of online activities was the next target of our interest. To investigate the extent of interactive Internet use for health-related communication, the following questions were formulated: “How often do you use the Internet to: 1) Interact with health professionals you have not met face-to-face?” 2) Participate in forum or self help groups (focusing on health or illness)? 3) Order medicines or other products related to health or illness management online?” and “Have you approached your family doctor, specialist, or other health professionals over the Internet (Web or e-mail)?” For the first three purposes, relevant frequency scales were provided grouped in “At least once a year” (“Every day,” “Every week,” “Every month,” “Every six months,” and “Every year”), “Less than once a year” and “Never” categories. For the fourth purpose, which concerned online interaction with well-known health professionals, dichotomous “Yes”/”No” responses were assessed. The questionnaire also contained items related to socio-demographic characteristics and health conditions (e.g., respondent’s age, gender, education, place of residence, frequency of doctor appointments, and overall health state) (see Additional file 1).

Respondents were provided with comprehensive information about the objectives and scope of the survey and gave their informed consent. The survey protocol was approved by the Bioethical Committee at the Wroclaw Medical University (statutory activity 481/2010).

### Data analysis

Statistical analysis was performed on the total number of 3027 participants aged 15–80+ from 2005, 2007, and 2012. The analysis mainly compared change in proportions from 2005 to 2007 and 2012. Two quantitative variables (age and numbers of doctor’s visits during the previous 12 months), which had no normal distribution, were verified using the Shapiro-Wilk test of normality. Arithmetic mean, standard deviation, median, as well as the range of variability (extremes) were calculated for quantitative variables, whereas a frequency (percentage) with a 95% confidence interval was determined for qualitative variables. To assess the distribution of the quantitative variables in main groups (Internet Users and Internet Non-Users, Internet Users for Health Purposes and Internet Non-Users for Health Purposes), the nonparametric Wilcoxon rank order test for unrelated samples was conducted. For qualitative variables, the chi-square test was used to determine statistically significant dependencies. Comparisons were performed separately for respondents from each year of the study (2005, 2007, and 2012) and in total, i.e., from among all of them. The significance level was set at p = .05. In addition to statistical significance, estimates of effect size (EF), which is the difference in location for quantitative variables, and Cramer’s V, which represents qualitative variables and their confidence intervals, were calculated. Effect size provides a description of the size of observed effects that is independent of the possibly misleading influences of sample size. Statistical analysis was carried out via R version 3.0.2 software.

Subsequently, correspondence analysis was conducted. This method provides information similar to the interpretation of the results of factor analysis, but on qualitative variables. Analysis of statistics and charts–proposed by this method–allows simple and intuitive inference about relationships occurring between categories of variables. With the help of the correspondence analysis, the profile of the most likely Internet user, and the person for whom the Internet is an important source of health information, was determined. Profiles were defined separately for respondents from each year of the study (2005, 2007, and 2012).

Each of the variables used in the analysis of correspondence was first converted to a 2- or 3-categorial feature to ensure the best subsequent interpretation of the clusters on a 2-dimensional graph. As a result of correspondence analysis carried out for a set of *n* 2-categorial and *m* 3-categorial variables, a two-dimensional graph is obtained, which is a set of *2n* + *3m* points. Each point corresponds to one category. The set of points can form clusters, i.e., subsets of points located closer to each other. The correspondence analysis method is rooted in the fact that the categories (points) belonging to clusters are interpreted as related to each other.

## Results

A total of 3027 people randomly selected from the Polish population were included in the analysis. The study group consisted of 1454 males (48%) and 1573 females (52%). The median age was 43 years (min-max: 15–94). With respect to location, 31.9% of respondents (n = 966) lived in big cities (above 100.000 residents), 31.1% (n = 941) in minor cities, and 37% in rural areas (n = 1120). As far as employment status is concerned, 44.9% (n = 1359) of the respondents had paid work, 13.5% (n = 409) were still in education, 28.8% (n = 872) were retired or did housework, and 4.8% (n = 145) were permanently sick or disabled. Further information about the study population is provided in Additional file 2.

### General use of the Internet compared with health-related purposes and demographics

**Figure 1:**
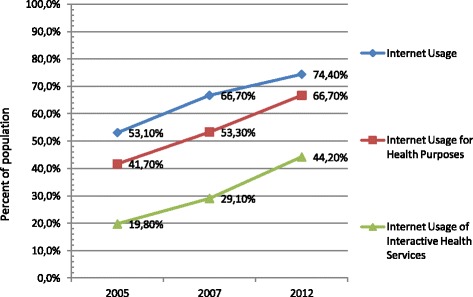
**Trends in Internet usage in years 2005–2007–2012: Internet usage, Internet usage for health purposes, Internet usage of interactive health services.**

The number of IU considerably increased from 53.1% in 2005, to 66.7% in 2007, and to 74.4% in 2012. The percentage of the Polish population that used the Internet for health purposes also increased by the same ratios (Figure 1). The essential growth of the Internet Users for Health Purposes (IUHP) was observed not only in the general population but also among the subgroup of IU accounting for 78.5% in 2005, 79.9% in 2007, and reaching 89.7% in 2012. The same trend concerned Internet usage of interactive health services (37.2% in 2005, 43.6% in 2007, and 59.4% of IU in 2012).

**Figure 2:**
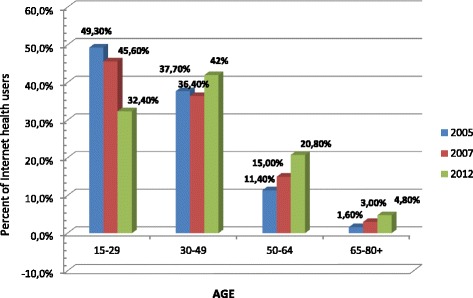
**Internet health usage by age categories in years 2005–2007–2012.**

Taking into consideration age categories, in the youngest age group (aged 15–29 years) the significant declining trend was observed and the number of IU decreased from 47.3% in 2005 to 30.8% in 2012. In category of middle age people (aged 30–49 years) the slightly increasing trend was observed (38% in 2005 and 41.8% in 2012), and this group finally became the largest group among IU in 2012. Despite the fact that groups of older people were in a minority in the total population of IU, it has been observed in these groups the most significant rising trend: the number of IU increased almost twofold in people aged 50–64 years and threefold in the oldest age category (see Additional file 3: Table S1). The same trend was found in the subgroup of IUHP: a considerably increasing number of elderly people at the expense of the youngest age category (Figure 2, Additional file 3: Table S2).

**Figure 3:**
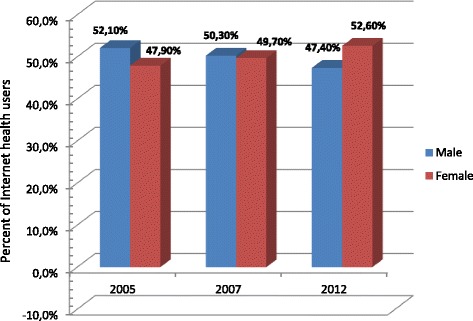
**Internet health usage by gender in years 2005–2007–2012.**

As far as gender comparison is concerned, in 2005 men slightly predominated in IUHP, but with the passage of time the differences quickly began to disappear. Finally, in 2012 women were at a slight advantage (Figure 3) with the exception of the oldest age group, where men were still prevalent. The same tendency was observed in the subgroup of IU but it was not as clear (Additional file 4: Table S3 and Table S4).

### Sources of information about health and illness

Throughout the study, face-to-face interaction with a family physician, other health professionals, and family and friends remained the most required source of medical information, occupying leading positions in the ranking. However, the Internet was one of the less important sources of health information: from 2005 a significant increase of 6.1% was observed, and in 2012 the Internet was characterized as important by 49.4% of Polish citizens. Internet moved from seventh position in 2005 (mean score 2.98 +/- 1.66) to fifth position in 2012 (3.37 +/- 1.62) on the list of sources of medical information (Table 1). Recommendations received from pharmacists were also a fairly important source (3.47 +/- 1.36 in 2012), preceded only by health professionals, family and friends, and medical books. The sharpest declines were observed in the case of TV/radio, which ranking dropped from third position in 2005 to sixth in 2012, (3.9 +/- 1.11 in 2005 and 3.32 +/- 1.32 in 2012, respectively). These declines were also seen in newspapers and magazines, where rankings fell from fifth to seventh position (3.68 +/- 1.17 in 2005 and 3.21 +/- 1.3 in 2012, respectively). The least important source of information about health and illness accounted for courses and lectures, which took the last position during the studied period.

**Table 1:** **Importance of various sources of health information (5-point scale of importance, where 1 stands for ‘not important’ and 5 stands for very important’)**

**Sources of health information**	**2005**	**2005**	**2007**	**2007**	**2012**	**2012**
	**Mean (+/-SD)**	**RANK**	**Mean (+/-SD)**	**RANK**	**Mean (+/-SD)**	**RANK**
Internet	2.98 +/- 1.66	7	3.37 +/- 1.63	7	3.37+/-1.62	5
TV/radio	3.9 +/- 1.11	3	3.53 +/- 1.23	4	3.32+/- 1.32	6
Books, medical encyclopaedias, leaflets	3.84 +/- 1.29	4	3.69+/- 1.31	3	3.61+/- 1.34	3
Courses and lectures	2.82 +/- 1.6	8	2.76 +/- 1.62	8	2.96+/- 1.69	8
Newspapers, magazines	3.68 +/- 1.17	5	3.42+/-1.25	6	3.21+/- 1.3	7
Family, friends and collegues	3.98+/- 1.17	2	3.93+/- 1.22	2	3.89+/-1.21	2
Pharmacies	3.55+/- 1.36	6	3.43/- 1.36	5	3.47+/- 1.36	4
Direct face-to-face contact with health professionals	4.33 +/- 1.12	1	4.0+/- 1.3	1	4.15+/-1.22	1

### Patterns of the Internet use for health purposes

Searching for information about health or illness was a dominant activity of medical IU and involved 67% of IU in 2005, then rising to 80.8% of IU in 2012. A clear growing trend concerning interactive use of the Internet for health purposes was also observed (Figure 1). The percentage of consumers participating in forums or self-help groups at least once a year significantly increased from 14.9% of IU in 2005, to 20.4% of IU in 2007, to 24.1% of IU in 2012. Ordering medicine or other health products via the Internet had a similar increasing trend (from 7.2% of IU in 2005 to 18.8% of IU in 2012). The study also showed that people were increasingly willing to take part in online mutual communication with various health professionals including their family doctors and those who were unknown. The frequency of interacting with health professionals one has not met face-to-face at least once a year increased sharply among IU from 14.3% in 2005 to 30% in 2012. Online communication with the well-known family doctor, specialists, or other members of medical personnel was not as frequent but showed a continuous ascending trend (2.2% of IU in 2005, 4.3% of IU in 2007, 9.0% of IU in 2012). Taking into consideration all kinds of interactive health services using at least one of the above, the number of consumers increased by 51.3% of IU in 2012 (Table 2).

**Table 2:** **Percentage of consumers (in general, Internet Users, Internet Users for Health Purposes) who are using interactive Internet health services at least once a year**

**E-health activity**	**2005**	**2005**	**2005**	**2007**	**2007**	**2007**	**2012**	**2012**	**2012**
	**TOTAL N =1027**	**AMONG IU N = 545**	**Among IUHP N = 428**	**TOTAL N =1000**	**AMONG IU N = 667**	**Among IUHP N = 533**	**TOTAL N =1000**	**AMONG IU N = 744**	**Among IUHP N = 675**
	**n % 95% CI**	**% 95% CI**	**% 95% CI**	**n % 95% CI**	**% 95% CI**	**% 95% CI**	**n % 95% CI**	**% 95% CI**	**% 95% CI**
Interact with health professional you have not met face-to-face	78			108			223		
	7.6	14.3	18.2	10.8	16.2	20.3	22.3	30.0	33.0
	6.0 9.2	11.4 17.3	14.6 21.9	8.9 12.7	13.4 19.0	16.9 23.7	19.7 24.9	26.7 33.3	29.5 36.6
Participate in forum or self help groups	81			136			179		
	7.9	14.9	18.9	13.6	20,4	25.5	17.9	24.1	26.5
	6.2 9.5	11.9 17.8	15.2 22.6	11.5 15.7	17.3 23.4	21.8 29.2	15.5 20.3	21.0 27.1	23.2 29.8
Order medicines or other health products	39			77			140		
	3.8	7.2	9.1	7.7	11.5	14.4	14.0	18.8	20.7
	2.6 5.0	5.0 9.3	6.4 11.8	6.0 9.3	9.1 14.0	11.5 17.4	11.8 16.2	16.0 21.6	17.7 23.8
Approach your family doctor, specialist or other health professionals over the Internet	12			29			67		
	1.2	2.2	2.8	2.9	4.3	5.4	6.7	9.0	9.9
	0.5 1.8	1.0 3.4	1.2 4.4	1.9 3.9	2.8 5.9	3.5 7.4	5.2 8.2	6.9 11.1	7.7 12.2
Using at least one of the interactive services above	159			229			382		
	15.5	29,2	37.1	22.9	34.3	43.0	38.2	51.3	56.6
	13.3 17.7	25.5 33.1	32.6 41.7	20.3 25.5	30.7 37.9	38.8 47.2	35.2 41.2	47.8 54.9	52.9 60.3

### Factors affecting Internet usage and Internet health usage

Multivariate analysis of different factors influencing the general use of the Internet and its use for health purposes was conducted for 2005, 2007, and 2012. Regarding the general use of the Internet, a number of factors proved to be significant: age, gender, education, employment status, place of residence, general health status, frequency of visiting doctors, long-term illnesses or disability, and mobile phone use (Table 3). These factors were relevant during the first survey in 2005 and remained significant throughout the period of analysis to 2012. Among the respondents, IU were significantly younger than Internet Non-Users (INU) (p < .001). There were significantly more men among IU than women; however, this difference was beginning to fade during the study period (56% of men versus 44% of women in 2005; 50.4% of men and 49.6% of women in 2012). Furthermore, IU were better educated than INU (p < .001). Significantly more IU had higher education levels (C-level), and significantly more INU had primary/vocational education (A-level). Significantly more IU were employed or in education, while significantly fewer were retired/did housework (p < .001); considerably fewer respondents were permanently sick or disabled. With regard to place of residence, significantly more IU lived in big cities, while significantly more INU lived in rural areas (p < .001); however, the effect size seems to be weak in this regard (EF 0.15). As far as health status is concerned, more IU than (INU) assessed their general state of health as good/very good over the study period (p < .001). Moreover, IU significantly less frequently visited a doctor for the last 12 months (p < .001) and fewer IU suffered from long-term illnesses or disability (p < .001). Finally, IU used mobile phones significantly more often (96.1% of IU compared to 69% INU; p < .001 in 2012).

**Table 3:** Factors affecting Internet use in 2005–2007–2012 and in general*

**Characteristics**	**2005**	**2005**		**2007**	**2007**		**2012**	**2012**		**TOTAL**	**TOTAL**	
	**IU % (N = 545)**	**INU% (N = 482)**	P VALUE ES*** 95%CI	**IU% (N = 667)**	**INU% (N = 333)**	P VALUE ES*** 95%CI	**IU% (N = 744)**	**INU% (N = 256)**	P VALUE ES*** 95%CI	**IU% (N = 1956)**	**INU% (N = 1071)**	P VALUE ES*** 95%CI
Sex:												
Men	56.0	39.2	**<0.001**	54.1	36.9	**<0.001**	50.4	38.9	**0.002**	53.2	38.4	**<0.001**
Women	44.0	60.8	**0.17**	45.9	63.1	**0.16**	49.6	61.1	**0.10**	46.8	61.6	**0.14**
			**0.1 0.23**			**0.1 0.22**			**0.04 0.16**			**0.11 0.18**
Age (years)												
Mean	33.63	52.24	**<0.001**	35.81	55.99	**<0.001**	39.59	64.58	**<0.001**	36.64	56.32	**<0.001**
±SD	±13.88	±15.62		±14.6	±14.12		±14.57	±12.86		±14.5	±15.33	
Median	30	54	**-20**	33	57	**-22**	38	64.5	**-26**	35	57	**-21**
Min-max	15-79	15-80	**-22 -18**	15-80	15-80	**-24 -19**	18-91	21-94	**-28 -24**	15-91	15-94	**-22 -20**
Education**:												
A level	23.1	53.3	**<0.001**	22.2	55.3	**<0.001**	26.1	62.3	**<0.001**	23.9	56.1	**<0.001**
B level	43.9	37.3	**0.36**	53.5	39.0	**0.35**	36.7	27.8	**0.35**	44.4	35.6	**0.35**
C level	33.0	9.3	**0.29 0.42**	24.3	5.7	**0.29 0.41**	37.2	9.9	**0.28 0.41**	31.6	8.4	**0.32 0.39**
Employment status												
Paid work (including self-employment) and/or in education	80.7	31.3	**<0.001**	79.9	23.0	**<0.001**	69.9	17.5	**<0.001**	76.3	25.5	**<0.001**
Retired/housework/care for children or other persons/unemployed and others	17.4	56.0	**0.50**	18.0	70.1	**0.55**	28.2	74.1	**0.46**	21.7	64.7	**0.50**
Permanently sick or disabled	1.8	12.7	**0.44 0.56**	2.1	6.9	**0.49 0.61**	1.9	8.4	**0.4 0.52**	1.9	9.9	**0.46 0.53**
Residence: place												
Big cities (above 100000 residents)	36.7	24.3	**<0.001**	37.3	24.2	**<0.001**	34.8	23.6	**<0.001**	36.2	24.1	**<0.001**
Minor cities (below 100000 residents)	32.8	33.6	**0.15**	31.5	26.6	**0.18**	31 .0	28.0	**0.13**	31.7	30.1	**0.15**
Villages/rural area	30.5	42.1	**0.08 0.21**	31.2	49.2	**0.12 0.24**	34.2	48.4	**0.07 0.19**	32.1	45.8	**0.11 0.18**
Health status												
Very good/good	73.2	35.7	**<0.001**	73.0	31.4	**<0.001**	67.1	29.4	**<0.001**	70.8	32.9	**<0.001**
Fair	23.5	51.2	**0.38**	23.2	55.3	**0.40**	28.8	53.6	**0.35**	25.4	53.1	**0.37**
Poor/very poor	3.3	13.1	**0.32 0.44**	3.7	13.3	**0.34 0.46**	4.2	17.1	**0.29 0.41**	3.8	14.1	**0.34 0.41**
Frequency of doctor`s visits (during last 12 months)												
Mean	4.58	7.29	**<0.001**	4.80	6.57	**<0.001**	5.46	9.72	**<0.001**	4.99	7.64	**<0.001**
±SD	±5.32	±8.39		±6.38	±7.46		±9.48	±15.64		±7.49	±10.41	
Median	3	4	**-1**	3	4	**-1**	3	6	**-2**	3	5	**-1**
Min-max	0-30	0-50	**-2 -1**	0-50	0-50	**-2 0**	0-99	0-99	**-3 -1**	0-99	0-99	**-2 -1**
Chronic diseases/disability												
Yes, I personally	6.2	14.3	**<0.001**	5.0	13.6	**<0.001**	9.3	19.6	**<0.001**	7.0	15.3	**<0.001**
Yes, a person close to me	29.0	24.5	**0.14**	27.9	32.9	**0.17**	35.5	24.8	**0.15**	31.1	27.2	**0.13**
No	64.8	61.2	**0.07 0.19**	67.1	53.5	**0.11 0.23**	55.2	55.6	**0.09 0.21**	61.9	57.5	**0.10 0.17**
Mobile phone use												
Yes	NO DATA						96.1	69.0	**<0.001**	96.1	69.0	**<0.001**
No							3.9	31.0	**0.38**	3.9	31.0	**0.38**
									**0.31 0.44**			**0.31 0.44**

*Significant differences between groups are bold.

**The item related to the education of the respondents included eleven options, from basic to university level, specific to the Polish education system. The eleven levels were collapsed into three categories according to the International Standard Classification of Education (ISCED): (A) education level lower than upper secondary; (B) education level including upper secondary to post-secondary non-tertiary; and (C) education level covering all levels according to ISCED higher than post-secondary non-tertiary.

***If p ≤ 0.05 effect size (ES) with 95% CI is provided: difference in location for quantitative variables and Cramer’s V for qualitative variables.

The different situation concerned factors influencing the use of the Internet for health purposes. Taking into consideration among IU the subgroups of IUHP and Internet Non-Users for Health Purposes (INUHP), only a few factors proved to be significant: age, gender, frequency of doctor’s visits, and long–term illnesses or disability, (Table 4). As in the case of general Internet use, IUHP were significantly younger than INUHP (p < .001); however, effect size indicates a weak association (EF = -4). With regard to gender, the relationship changed over time. In 2005 significantly more men (52.1%) than women (47.9%) were seen among IUHP; in 2007, the differences faded (50.3% versus 49.7%, respectively). Finally, in 2012 significantly more women than men used the Internet for health purposes (47.4% versus 52.6%, respectively, p < .001); at the same time, significantly more men were among INUHP. Taking into consideration frequency of visiting doctors, the opposite relationship was observed regarding general Internet usage. Respondents indicating IUHP significantly more often visited a doctor than INUHP (p < .001), and this relationship maintained throughout the research period. With respect to chronic diseases or disability, significant differences were not found among IUHP and INUHP in 2005, 2007 and 2012. However, considering the total group of respondents, IUHP significantly more frequently suffered from long-term illnesses/disability or such a person was close to them (p = .006). No significant associations were found between IUHP and INUHP with regard to place of residence, employment status, state of health, and mobile phone use (see Table 4).

**Table 4:** Factors affecting Internet use for health purposes in 2005–2007–2012 and in general*

**Characteristics**	**2005**	**2005**		**2007**	**2007**		**2012**	**2012**		**TOTAL**	**TOTAL**	
	**IUHP% (N = 428)**	**INUHP% (N = 117)**	P VALUE ES*** 95%CI	**IUHP% (N = 533)**	**INUHP% (N = 134)**	P VALUE ES*** 95%CI	**IUHP% (N = 667)**	**INUHP% (N = 77)**	P VALUE ES*** 95%CI	**IUHP% (N = 1628)**	**INUHP% (N = 328)**	P VALUE ES*** 95%CI
Sex:												
Men	52.1	70.1	**<0.001**	50.3	70.5	**<0.001**	47.4	78.1	**<0.001**	49.6	72.1	**<0.001**
Women	47.9	29.9	**0.14**	49.7	29.5	**0.16**	52.6	21.9	**0.18**	50.4	27.9	**0.17**
			**0.06 0.23**			**0.08 0.23**			**0.11 0.25**			**0.12 0.21**
Age (years)												
Mean	33.05	35.77	0.089	34.71	40.47	**<0.001**	38.73	46.59	**<0.001**	35.92	40.15	**<0.001**
±SD	±13.56	±14.84		±14.20	±15.47		±14.20	±15.92		±14.24	±15.83	
Median	30	33		31	42	**-6**	37	47	**-8**	34	40	**-4**
Min-Max	15-77	15-79		15-80	15-72	**-9 -3**	18-83	20-91	**-12 -4**	15-83	15-91	**-6 -2**
Education**:												
A level	23.1	23.1	0.991	21.8	24.0	0.604	24.4	41.1	0.007	23.2	27.6	0.173
B level	43.7	44.4		53.3	55.0		38.1	24.7	0.12	44.5	44.2	
C level	33.2	32.5		25.0	20.9		37.5	34.2	0.04 0.19	32.2	28.2	
Employment status												
Paid work (including self-employment) and/or in education	79.0	87.2	0.111	81.4	74.4	0.056	69.3	72.6	0.886	75.8	78.7	0.484
Retired/housework/care for children or other persons/unemployed and others	19.1	11.1		16.2	24.8		28.7	26.0		22.1	20.1	
Permanently sick or disabled	1.9	1.7		2.4	0.8		2.0	1.4		2.1	1.2	
Residence: place												
Big cities (above 100000 residents)	38.1	31.7	0.352	37.8	36.4	0.199	35.4	29.6	0.534	36.8	33.1	0.128
Minor cities (below 100000 residents)	32.7	33.3		32.6	26.4		31.2	31.0		32.1	30.0	
Villages/rural area	29.2	35.0		29.6	37.2		33.4	39.4		31.1	36.9	
Health status												
Very good/good	73.1	73.5	0.999	73.7	70.5	0.725	67.5	64.4	0.650	71.0	70.2	0.878
Fair	23.6	23.1		22.5	25.6		28.2	32.9		25.1	26.4	
Poor/very poor	3.3	3.4		3.8	3.9		4.3	2.7		3.9	3.4	
Frequency of doctor`s visits (during last 12 months)												
Mean	4.85	3.62	**0.002**	5.09	3.60	**0.001**	5.60	4.44	**0.032**	5.23	3.80	**<0.001**
±SD	±5.42	±4.83		±6.64	±5.17		±9.72	±7.17		±7.81	±5.57	
Median	3	2	**1.0**	3	2	**1.0**	3	2	**1.0**	3	2	**1.0**
Min-Max	0-30	0-24	**0.0 1.0**	0-50	0-25	**0.0 1.0**	0-99	0-45	**0.0 1.0**	0-99	0-45	**0.0 1.0**
Chronic diseases/disability												
Yes, I personally	6.8	4.3	0.564	4.9	5.5	0.293	9.6	6.8	0.066	7.3	5.3	**0.006**
Yes, a person close to me	29.4	27.4		28.7	21.8		36.3	24.7		32.0	24.5	
												**0.07**
No	63.8	68.3		66.4	72.7		54.1	68.5		60.7	70.2	**0.02 0.11**
Mobile phone use												
Yes	NO DATA						96.6	91.8	0.057	96.6	91.8	0.057
No							3.4	8.2		3.4	8.2	

*Significant differences between groups are bold.

**The item related to the education of the respondents included eleven options, from basic to university level, specific to the Polish education system. The eleven levels were collapsed into three categories according to the International Standard Classification of Education (ISCED): (A) education level lower than upper secondary; (B) education level including upper secondary to post-secondary non-tertiary; and (C) education level covering all levels according to ISCED higher than post-secondary non-tertiary.

***If p ≤ 0.05 effect size (ES) with 95%CI is provided: difference in location for quantitative variables and Cramer’s V for qualitative variables.

### Profiles of IU and citizens for whom the Internet is an important source of health information

**Figure 4:**
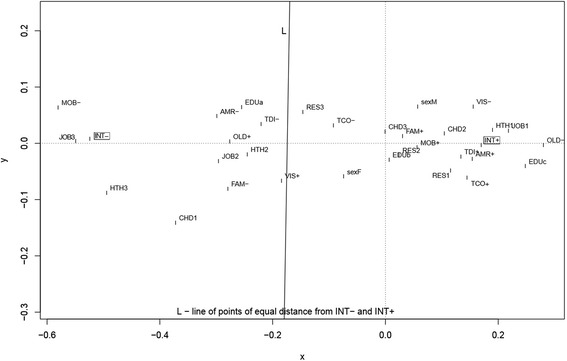
**Profile of Internet Users and Internet Non -Users (INT+/INT-) in year 2012 based on correspondence analysis (the legend in Additional file** **6** **).**

Correspondence analysis was used to draw the profiles of Internet Users and Non-Users (INT+/INT-) among the population in general and among those for whom the Internet is an important source of health information (SMI+/SMI-). The profiles were defined separately for respondents from each year of the study (2005, 2007, and 2012). As it was shown on a two-dimensional graph, several different variables belonged to the profile of Internet Users in 2012 (Figure 4). The most likely Internet user (INT+) was younger, had higher levels of education, had a paid job or was still in education, and lived in big cities. He subjectively assessed his health status as good or very good, less frequently visited doctors, and the person close to him usually suffered from chronic diseases. He also presented a positive attitude concerning teleconsultations, supported telediagnosis, and was willing to get access to his medical records. On the other hand, features that were strongly associated with a reluctance to use the Internet were: poor health, suffering from long-term illnesses, disability, and an inability to use a mobile phone. The set of variables did not differ significantly for the profiles of IU and INU in 2005 and 2007 (Additional file 5: Figure S1 and Figure S2). Minor differences especially concerned males, a feature associated with the profile in 2005, but over time this ceased to be a related factor.

**Figure 5:**
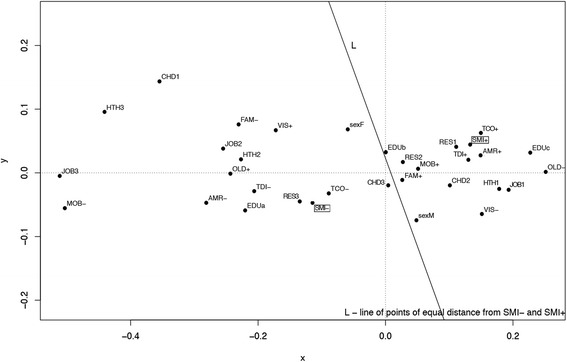
**Profile of persons who consider the Internet as an important or not important source of medical information (SMI+/SMI-) in year 2012 based on correspondence analysis (the legend in Additional file** **6** **).**

As previously mentioned, profiles of citizens for whom the Internet is an important or not important source of health information were also determined (Figure 5). The profile in 2012 did not differ significantly from the profiles in 2007 and 2012; the set of variables proved to be constant (Additional file 7: Figure S3 and Figure S4). It is also interesting to note that features describing SMI+/ SMI- profiles were very similar to the previously described profiles of INT+/INT-. The most significant variables determining the profile of SMI+ were: younger age, male gender, living in a big city, higher levels of education, paid work and/or continuing education, good health status, infrequent visits to the doctor, chronic diseases in relatives, and a positive attitude regarding teleconsultations, telediagnosis, and getting access to their own medical records (Figure 5).

## Discussion

### General use of the Internet compared with health-related purposes and demographics

The vast majority of the Polish population nowadays uses the Internet for health purposes. We can observe a significant increase of 25% usage from 2005 to 2012. The growth is consistent with the trends observed in both Europe and globally [9,11,13,33]. According to the Manhattan Research, a 30% increase in health-related usage of the Internet was seen for the four years ranging from 2004 to 2008 [34]. In turn, the Cybercitizen Health Survey showed that since 2002 the use of the Internet for activities seeking health information has steadily increased, reaching 72% of e-health consumers in 2011 [35]. It is also worth noting that in Polish surveys the growth in the number of IUHP is relatively larger than the growth due to the number of IU and cannot solely be explained by improved Internet access (IUHP accounted for 79.9% of IU in 2007 and 89.7% of IU in 2012). A similar situation for Denmark, Germany, Greece, and Portugal was reported by Kummervold et al. [11]. This may indicate that new Internet e-health services appeared on the consumer market and have become available via the Internet.

The main IU as well as IUHP are still younger people [10,11,23,26,36,37]. However, the elderly have the largest growth potential: this figure tripled during the study period. Strong growth in e-health adoption among older consumers is also confirmed by other authors [9,27,35]. Men’s predominance, observed in 2005, as IU and IUHP gradually disappeared, and in 2012 women reported more health-related usage, which is in line with many other studies [10,11,33,36-39]. While an overall growth in Internet health-related usage among women was seen, this did not apply to the oldest age category, where the increase was largest among men. This might be explained by the fact that elderly people are still first generation of IU, where male dominance is typical.

### Sources of information about health and illness

The significance of the Internet as a source of health information is growing. As the study showed, the Internet has become an important source of health information for almost half of Polish citizens ahead of TV/radio, newspapers/magazines, and courses/lectures in the ranking. A population-based survey on e-health trends in Europe from 2007 [11] revealed that the Internet was important source of health information for 46.8% of the population. Clearly leading in this respect were the Nordic countries, especially Denmark, for whom the Internet was the second most important source outranked only by information from health professionals. Other findings are even more encouraging. The international survey conducted in 2011 by Health On the Net Foundation showed the Internet as the second-ranked source of health information after physicians, due to its accessibility and “easiness of use” [40]. In turn, a recent cross-sectional survey conducted among Dutch population, has already found the Internet as the number one source for health-related information (82.7% of respondents) [41]. Furthermore, the Cybercitizen Health Survey reports that Internet currently has the greatest reach as a health information source. However, doctors, nurses, pharmacists, and other health care professionals have still the strongest influence on citizens’ health decisions [35].

### Patterns of the Internet use for health purposes

Searching for information about health and illness was definitely the most frequent activity performed online by respondents in the study and reflects their main interests in this area. Similar patterns of medically related Internet activities, with searching for health information as a dominant activity, were reported in many other studies concerning both general Internet use [9,10,13,15,21,22,26,42] and its use in various selected groups of population [19,27,28,43,44]. However, a tendency towards a more “advanced” usage of the Internet for health purposes is also found. In the study, the use of all interactive, health-related online services increased significantly, ending in a total of 44.2% of respondents in 2012. This trend had already been observed earlier by other authors [11,29,30]. Kummervold et al. [11] reported a growth of European consumers using interactive Internet health services at least once a year from 15.3% in 2005 to 22.7% in 2007. Additionally, online communication with doctors or other health professionals whom they have not previously met proved to be the leading choice among interactive Internet services, which is also confirmed by our survey.

The interactive use of the Internet for health purposes has been of particular interest in Poland in recent years. This can be partially explained by the growing dissatisfaction of the Polish society with a functioning health care system based on public hospital services and outpatient care provided by the private sector working mainly on a contract with the National Health Fund [18,45,46]. Deteriorating access to health care services, a queue system frequently forcing patients to wait months for certain specialist services, and a large, omnipresent bureaucracy force Polish citizens to seek medical help in online forums and make them eager to use various e-health services offered by private medical centers [10,19,20,47-49]. On the other hand, as IU become more experienced and comfortable with opportunities provided by the Web, they begin to use the Internet increasingly often as a useful communication channel with health professionals. New e-health technologies provide opportunities for more empowered patients, so-called e-patients who see themselves as equal partners with their doctors in the health care process [34,35,50,51]. The Cybercitizen Survey showed that 99 million adults in the United States are e-empowered patients and actions most often undertaken by them have been aimed at using the Internet instead of a doctor (61.1%), discussing information found online at appointments with health professionals (54.7%), and changing their health decisions based on information obtained online (45.8%) [35]. It seems that the trend can develop in the future, and physicians should be prepared for it.

### Factors affecting Internet usage and Internet health usage

A number of factors proved to be related to the general use of the Internet and its use for health purposes in 2005–2012. Regarding age and gender, as it has been mentioned, younger people and women were the most active Internet health users. In terms of education, higher levels of education strongly affected the use of the Internet; however, for health purposes the relationship was not so clear and significant only for the survey conducted in 2012. In the study, significantly more IU had paid work or were in education; similarly, in other surveys, studying [36], employment status [18,23,36,38], higher socio-cultural position [10,37], higher income, and stable income [18,26,39] were factors that significantly influenced both general Internet usage and its use for health-related activities. With respect to place of residence, citizens in urban locations (above 100.000 residents) more often used the Internet than inhabitants in rural areas. However, the relationship seemed to be weak, and when we took into consideration only the subgroup of IUHP, a digital division between urban and rural residents was not present, which was also confirmed by Duplaga [19]. As far as health status and medical issues are concerned, the findings differ depending on whether the group of IU or IUHP was taken into account. Better self-rated state of health proved to affect only general Internet use, and IU significantly less frequently visited a doctor and suffered from long-term illnesses or disability. On the contrary, no significant relationship was found between IUHP and health status, and IUHP significantly more often visited a doctor. Moreover, IUHP or person close to them more frequently suffered themselves from long-term illnesses/disabilities. Such a discrepancy is difficult to explain; however, it seems that the situation is associated with a predominance of females among IUHP that actively search Internet for health-related information, more frequently share their health problems with a doctor, and experience various chronic diseases themselves or in their family. Other authors have confirmed some of these issues [10,36,39,52-54], but the studies in this area should certainly be continued. Finally, in what has been confirmed by several other studies [9,26,49], IU used mobile phones significantly more often; this association can be observed also among IUHP but dependency is weaker and without the statistical significance.

In most cases, all relationships were stable and did not change during the study period (2005–2012), which indicates their strong and well-established connection with the use of the Internet. The exception, as mentioned earlier, is the gender feature: in this case, male domination was replaced by female in health-related information seeking during the study period.

### Profiles of IU and citizens for whom the Internet is an important source of health information

Based on correspondence analysis, profiles of the most likely Internet user and the citizen for whom the Internet is an important source of health information were constructed. The most significant variables determining both profiles in 2012 were: younger age, higher level of education, living in a big city, paid work and/or continuing education, good health status, infrequent visits to the doctor, chronic diseases in relatives, and a positive attitude regarding teleconsultations, telediagnosis, and getting access to their own medical records. The findings are to some extent consistent with the results presented by Santana et al. [31]. In seven European countries participating in the study, the citizen using the Internet to get health information to help deal with the consultation was most likely to appear as someone young, with higher education, living in a big city, consulting at least once with a doctor the previous year, and using the Internet frequently to read health websites, order medicine, participate in forums, and on an infrequent basis, to interact with a health professional they had never before met face-to-face. In turn, the study comparing e-health consumer attitudes in Poland and Greece [55] showed that, regarding teleconsultation, the acceptance of medical televisits was still low in citizens in both countries; much more positive attitudes were related to telediagnosis and online access to their own medical records. In particular, which corresponds with our profiles, acceptance appeared to be wider among IU in general and for health purposes.

### Limitations

The study has several limitations. The method of collecting data used in the study was CATI, so some variables seemed to be difficult to include in the survey. One of them was income. It would have been inappropriate to ask about income during a telephone interview. Furthermore, there were few variables such as telemonitoring, telediagnosis, online access to medical records, and mobile phone use that were not included in the first survey in 2005 and even in the second survey in 2007 (mobile phone use). So in these cases, it was not possible to fully observe how and if these characteristics are subject to change over time and the corresponding designation of the trend line.

The average response rate of 33.5%, as is usual for this type of study, was low, so it could cause “non-response” and “non-coverage” bias and affect the estimates that were made. In Poland in 2005–2012, average telephone penetration was 74% (taking into consideration both landlines and mobile phones) but one should be aware that the utilization of telecommunication instruments is usually much lower among people living in rural areas, having low education, and being of advanced age.

The method of sample collection*,* reporting the data, and analyzing the data were exactly the same during all three waves of the study; however, the study did not follow the same individuals over the research period. So changes observed in the use, perception, and preferences regarding the Internet and, in particular, its use for health purposes, can be analyzed only from the perspective of the general population or distinguished subgroups and cannot represent the attitudes and opinions of individual participants in the study.

Although some of these results have already been reported in the published literature, the study complements preceding surveys significantly. It investigates trends and changing patterns of Polish health-related Internet use over the past seven years. To the best of our knowledge, it is the first Polish long-term study in this field.

## Conclusions

As it has been shown in the study, in Poland in 2005–2012 we could observe a sharply rising trend in both the total number of IU and IUHP. The significance of the Internet as a source of health information has also grew. The Internet has become an important source of health information for almost half of Polish citizens, overtaking TV/radio, newspapers/magazines, and courses/lectures in the ranking list. As the medium matures, the use of interactive, health-related online services has increased significantly as well. Interacting via the Internet with health professionals one has not met face-to-face proved to be the most frequently used online health activity. Interactivity and the large amount of information available over Internet empower citizens to take better care of themselves and to communicate with their doctors. The number of women as Internet Users for Health Purposes continues to grow. While the main users of the Internet are certainly younger people, the study indicates that the largest growth potential was observed to be among the elderly. There are several factors influencing the general use of the Internet and for health-related matters. This has made it possible to outline both the profile of the most likely Internet user as well as the citizen for whom the Internet is an important source of health information.

The Internet offers enormous opportunities, particularly for offering and improving consumer information services with regard to health care. The conducted study has been able to show some trends over the seven-year period. It would certainly be vital and interesting to follow up on the research in the coming years to find what the pace and direction of further changes will be, whether the upward trend still continues, and which Internet services will become the most desirable and fastest growing among Polish citizens.

## Additional files

Additional file 1:
**E-Health consumer trend survey 2012 Questionnaire.**
Additional file 2:
**Selected demographic characteristics and health conditions.**
Additional file 3: Table S1. Internet usage by age and gender. **Table S2.** Internet health usage by age and gender. Additional file 4: Table S3. Internet usage by gender. **Table S4.** Internet health usage by gender. Additional file 5: Figure S1. Profile of Internet Users and Internet Non Users (INT+/INT-) in year 2005 based on correspondence analysis. **Figure S2.** Profile of Internet Users and Internet Non Users (INT+/INT-) in year 2007 based on correspondence analysis. Additional file 6:
**Collective legend for Figures** 4 **and** 5 **and Additional files** 5 **and** 7 **.**
Additional file 7: Figure S3. Profile of persons who consider the Internet as an important or not important source of medical information (SMI+/SMI-) in year 2005 based on correspondence analysis. **Figure S4.** Profile of persons who consider the Internet as an important or not important source of medical information (SMI+/SMI-) in year 2007 based on correspondence analysis.

## Abbreviations

ICT: Information and communication technology
CATI: Computer-assisted telephone interview
EF: Effect size
IU: Internet Users
INU: Internet Non-Users
IUHP: Internet Users for Health Purposes
INUHP: Internet Non-Users for Health Purposes
