# Supplementary material for: Trends in the use of the Internet for health purposes in Poland
Source: BMC Public Health. 2015 Feb 27;15:194. doi: 10.1186/s12889-015-1473-3 (PMC4349300; doi:10.1186/s12889-015-1473-3)
Supplement: Additional file 2: — Selected demographic characteristics and health conditions. [file 12889_2015_1473_MOESM2_ESM.pdf]

Additional file 2   Selected demographic characteristics and health conditions

CHARACTERISTICS	ALL RESPONDENTS N =3027	ALL RESPONDENTS %
SEX: MEN<br>WOMEN	1454<br>1573	48,0<br>52,0
AGE<br>Years (+/- SD)<br>Median (min-max)	 43,61 (+/- 17,5)<br>43 (15-94)	
EDUCATION*:<br>A level<br>B level<br>C level	 1065<br>1251<br>711	 35,18<br>41,33<br>23,49
EMPLOYMENT STATUS<br>Paid work (including self-employment)<br>In education<br>Unemployed<br>Retired<br>Housework/care for children or other persons<br>Permanently sick or disabled<br>Other	 1359<br>409<br>224<br>681<br>191<br>145<br>18	 44,9<br>13,5<br>7,4<br>22,5<br>6,3<br>4,8<br>0,6
RESIDENCE: PLACE<br>Big cities (above 100000 residents)<br>Minor cities<br>Villages/rural area	 966<br>941<br>1120	 31,9<br>31,1<br>37,0
HEALTH STATUS (subjective assessment)<br>Very good<br>Good<br>Fair<br>Poor<br>Very poor	 539<br>1204<br>1068<br>176<br>40	 17,8<br>39,8<br>35,3<br>5,8<br>1,3
FREQUENCYOF DOCTOR`S VISITS (during last 12 months)<br>N (mean +/- SD)<br>Median (min-max)	 5,91 (+/- 8,54)<br>3 (0-99)	
CHRONIC DISEASES/DISABILITY<br>Yes, I personally<br>Yes, a person close to me<br>No	 402<br>978<br>1647	 13,3<br>32,3<br>54,4

* The item related to the education of the respondents included eleven options, from basic to university level, specific to the Polish education system. The eleven levels were collapsed into three categories according to the International Standard Classification of Education (ISCED): (A) education level lower than upper secondary; (B) education level including upper secondary to post-secondary non-tertiary; and (C) education level covering all levels according to ISCED higher than post-secondary non-tertiary.
