# Supplementary material for: Trends in the use of the Internet for health purposes in Poland
Source: BMC Public Health. 2015 Feb 27;15:194. doi: 10.1186/s12889-015-1473-3 (PMC4349300; doi:10.1186/s12889-015-1473-3)
Supplement: Additional file 3: Table S1. — Internet usage by age and gender. Table S2. Internet health usage by age and gender. [file 12889_2015_1473_MOESM3_ESM.pdf]

Table S1 Internet usage by age and gender

| INTERNET USAGE | 2005 (N=545) % (CI) | 2007 (N =667) % (CI) | 2012 (N=744) % (CI) |
|---|---|---|---|
| Age categories: 15 -29 | 47.3 (43.1-51.5) | 42.3 (38.5-46.0) | 30.8 (27.5-34.1) |
| Male | 24.9 (21.3-28.6) | 21.6 (18.5-24.7) | 15.7 (13.1-13.8) |
| Female | 22.4 (18.9-25.9) | 20.7 (17.6-23.8) | 15.1 (12.5-17.6) |
| Age categories: 30-49 | 38.0 (33.9-42.1) | 37.9 (34.2-41.6) | 41.8 (38.3-45.3) |
| Male | 21.5 (18.0-24.9) | 21.0 (17.9-24.1) | 21.1 (18.2-24.0) |
| Female | 16.5 (13.4-19.6) | 16.9 (14.1-19.8) | 20.7 (17.8-23.6) |
| Age categories: 50-64 | 12.5 (9.7-15.3) | 16.1 (13.3-18.8) | 22.0 (19.1-25.0) |
| Male | 8.1 (5.8-10.4) | 9.3 (7.1-11.5) | 10.4 (8.2-12.5) |
| Female | 4.4 (2.7- 6.1) | 6.8 (4.8- 8.7) | 11.6 (9.3-13.9) |
| Age categories: 65-80+ | 2.2 (1.0-3.4) | 3.7 (2.3-5.2) | 5.4 (3.8-7.0) |
| Male | 1.5 (0.5-2.5) | 2.2 (1.1-3.4) | 3.1 (1.8-4.3) |
| Female | 0.7 (0.0-1.5) | 1.5 (0.6-2.4) | 2.3 (1.2-3.4) |

Table S2 Internet health usage by age and gender

| INTERNET HEALTH USAGE | 2005 (N=428) % (CI) | 2007 (N=533) % (CI) | 2012 (N=667) % (CI) |
|---|---|---|---|
| Age categories: 15 -29 | 49.3 (44.6-54.0) | 45.6 (41.4-49.8) | 32.4 (28.8-35.9) |
| Male | 24.3 (20.2-28.4) | 21.2 (17.7-24.7) | 15.9 (13.1-18.7) |
| Female | 25.0 (20.9-29.1) | 24.4 (20.7-28.0) | 16.5 (13.7-19.3) |
| Age categories: 30-49 | 37.7 (33.0-42.2) | 36.4 (32.3-40.5) | 42.0 (38.2-45.7) |
| Male | 19.7 (15.9-23.4) | 18.9 (15.6-22.3) | 20.2 (17.2-23.3) |
| Female | 18.0 (14.4-21.6) | 17.5 (14.2-20.7) | 21.8 (18.6-24.9) |
| Age categories: 50-64 | 11.5 (8.4-14.5) | 15.0 (12.0-18.0) | 20.8 (17.8-23.9) |
| Male | 6.8 (4.4- 9.2) | 8.3 (5.9-10.6) | 8.5 (6.4-10.7) |
| Female | 4.7 (2.7- 6.7) | 6.7 (4.6- 8.9) | 12.3 (9.8-14.8) |
| Age categories: 65-80+ | 1.6 (0.4-2.8) | 3.0 (1.6-4.5) | 4.8 (3.2-6.4) |
| Male | 1.4 (0.3-2.5) | 1.9 (0.7-3.0) | 2.7 (1.5-3.9) |
| Female | 0.2 (0.0-0.7) | 1.1 (0.2-2.0) | 2.1 (1.0-3.2) |
