# Supplementary material for: Trends in the use of the Internet for health purposes in Poland
Source: BMC Public Health. 2015 Feb 27;15:194. doi: 10.1186/s12889-015-1473-3 (PMC4349300; doi:10.1186/s12889-015-1473-3)
Supplement: Additional file 4: Table S3. — Internet usage by gender. Table S4. Internet health usage by gender. [file 12889_2015_1473_MOESM4_ESM.pdf]

Table S3 Internet usage by gender

INTERNET USAGE	2005 (N=545)<br>n<br>%<br>95%CI	2007 (N =667)<br>n<br>%<br>95%CI	2012 (N=744)<br>n<br>%<br>95%CI
Sex: Male	305<br>56,0<br>51,8 60,1	361<br>54,1<br>50,3 57,9	375<br>50,4<br>46,8 54,0
Sex: Female	240<br>44,0<br>39,9 48,2	306<br>45,9<br>42,1 49,7	369<br>49,6<br>46,0 53,2

Table S4 Internet health usage by gender

INTERNET HEALTH USAGE	2005 (N=428)<br>n<br>%<br>95%CI	2007 (N=533)<br>n<br>%<br>95%CI	2012 (N=667)<br>n<br>%<br>95%CI
Sex: Male	223<br>52,1<br>47,4 56,8	268<br>50,3<br>46,0 54,5	316<br>47,4<br>43,6 51,2
Sex: Female	205<br>47,9<br>43,2 52,6	265<br>49,7<br>45,5 54,0	351<br>52,6<br>48,8 56,4
