# Supplementary material for: Trends in the use of the Internet for health purposes in Poland
Source: BMC Public Health. 2015 Feb 27;15:194. doi: 10.1186/s12889-015-1473-3 (PMC4349300; doi:10.1186/s12889-015-1473-3)

Figure S1 Profile of Internet Users and Internet Non Users (INT+/INT-) in 2005 based on correspondence analysis

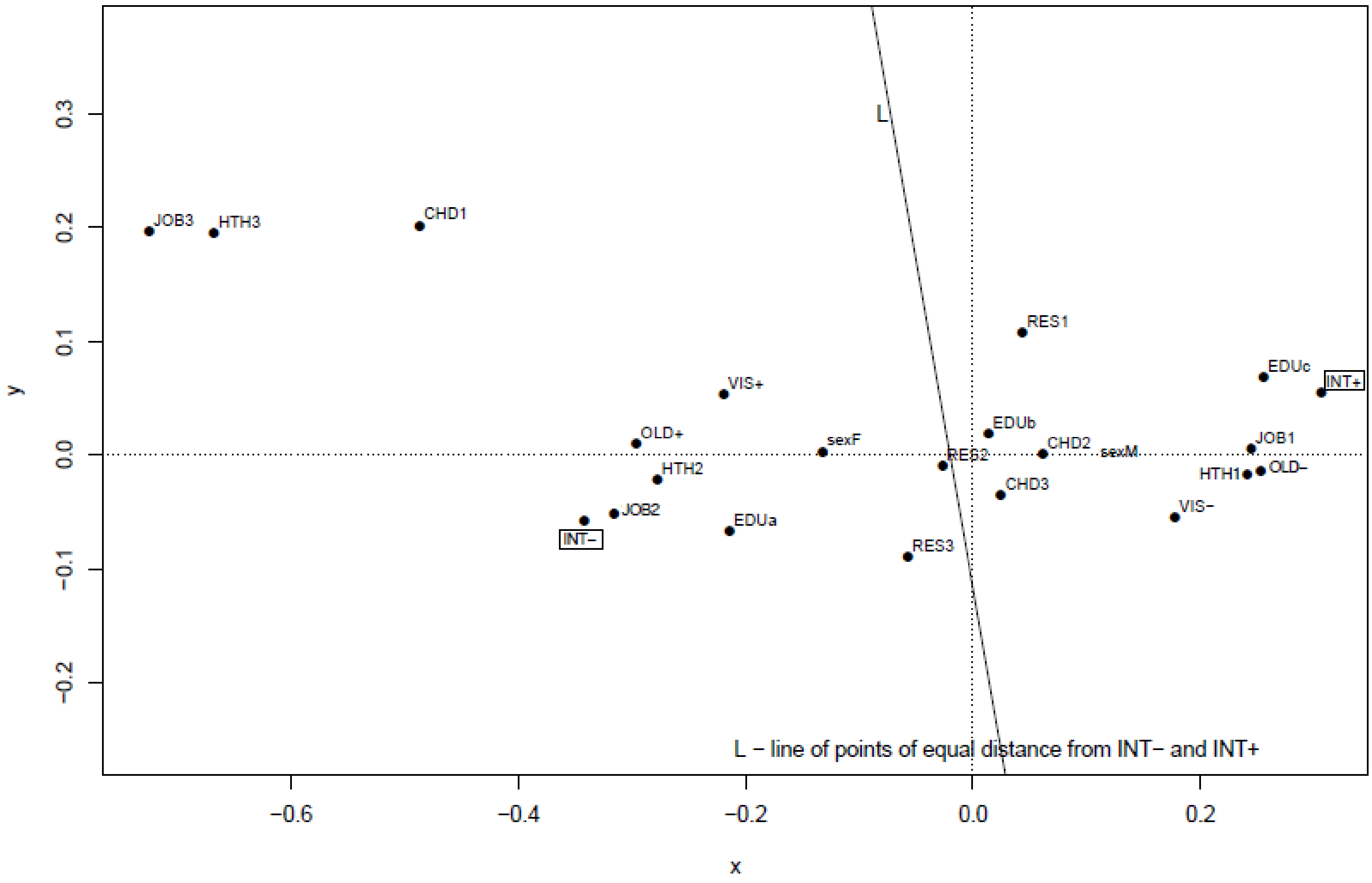

Figure S2 Profile of Internet Users and Internet Non Users (INT+/INT-) in 2007 based on correspondence analysis

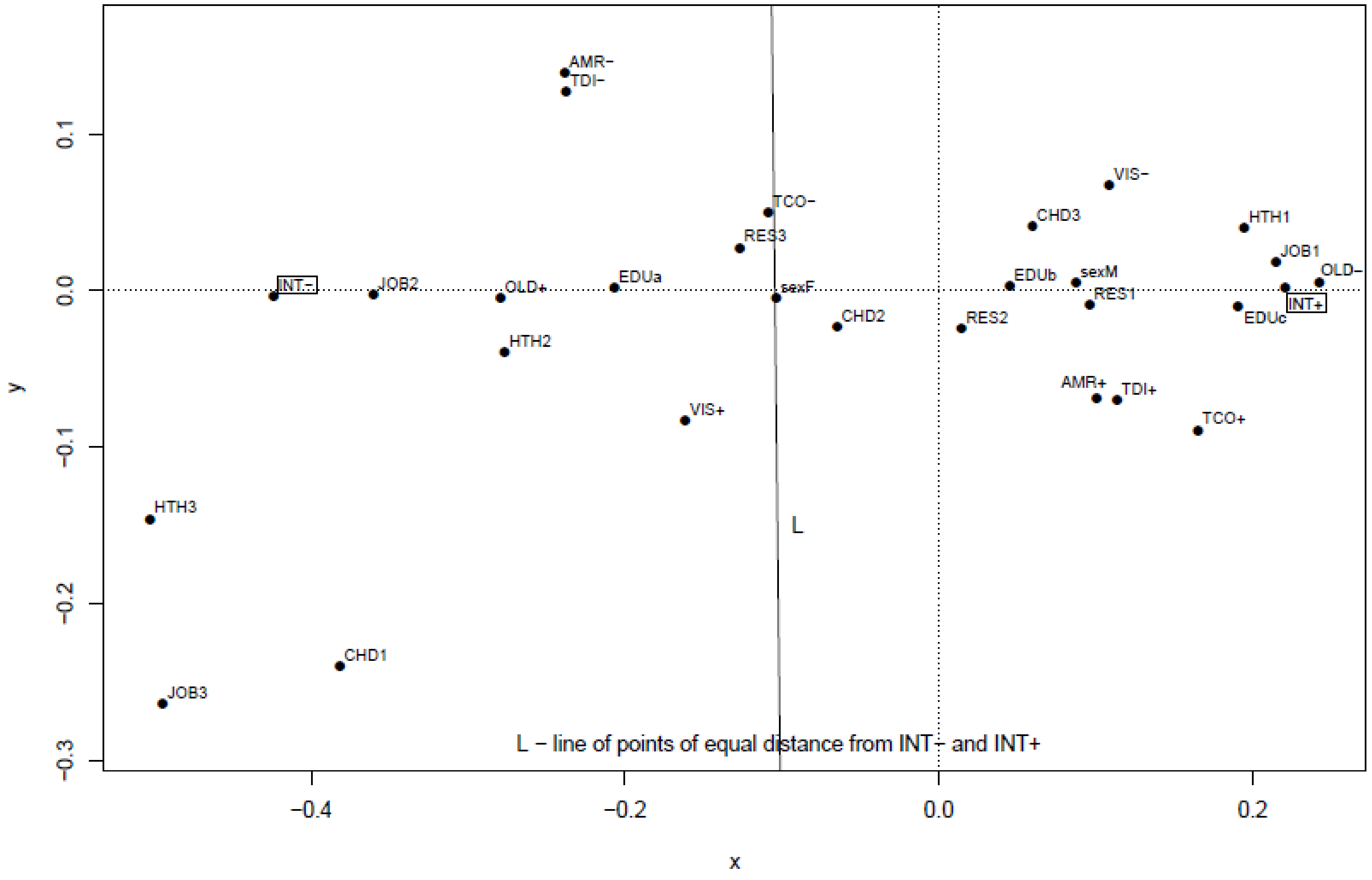

Supplement: Additional file 5: Figure S1. — Profile of Internet Users and Internet Non Users (INT+/INT-) in year 2005 based on correspondence analysis. Figure S2. Profile of Internet Users and Internet Non Users (INT+/INT-) in year 2007 based on correspondence analysis. [file 12889_2015_1473_MOESM5_ESM.pdf]
