# Supplementary material for: Trends in the use of the Internet for health purposes in Poland
Source: BMC Public Health. 2015 Feb 27;15:194. doi: 10.1186/s12889-015-1473-3 (PMC4349300; doi:10.1186/s12889-015-1473-3)
Supplement: Additional file 6: — Collective legend for Figures 4 and 5 and Additional files 5 and 7 . [file 12889_2015_1473_MOESM6_ESM.doc]

| Variable | Code | Description |
| --- | --- | --- |
| Year | 2005 |  |
|  | 2007 |  |
|  | 2012 |  |
| Age | OLD- | Age < 43 |
|  | OLD+ | Age > 42 |
| Sex | sexM | Male |
|  | sexF | Female |
| Education | EDUa | A level |
|  | EDUb | B level |
|  | EDUc | C level |
| Residence: type | FAM- | Alone |
|  | FAM+ | With family |
| Residence: place | RES1 | Big cities (above 100000 residents) |
|  | RES2 | Minor cities (below 100000 residents) |
|  | RES3 | Villages / rural area |
| Employment status | JOB1 | Paid work (including self-employment) and/or in education |
|  | JOB2 | Retired / housework / care for children or other persons / unemployed and others) |
|  | JOB3 | Permanently sick or disabled |
| Internet use | INT- | Internet No Use |
|  | INT+ | Internet Use |
| Frequency of doctor's visits | VIS- | Frequency < 4 |
|  | VIS+ | Frequency > 3 |
|  |  |  |
| Chronic diseases/ disability | CHD1 | Yes, personally |
|  | CHD2 | Yes, a person close to me |
|  | CHD3 | No |
| Health status | HTH1 | Very good / good |
|  | HTH2 | Fair |
|  | HTH3 | Poor / very poor |
| Teleconsultations | TCO- | Opposite to teleconsultations / Undecided |
|  | TCO+ | Supporting t eleconsultations |
| Telediagnosis | TDI- | Opposite to telediagnosis / Undecided |
|  | TDI+ | Supporting telediagnosis |
| Access to the medical records | AMR- | Unwilling for access to the medical records / Undecided |
|  | AMR+ | Willing for access to the medical records |
| Mobile phone use | MOB- | No |
|  | MOB+ | Yes |
| Internet as a source of medical information | SMI- | Not important |
|  | SMI+ | Important |
