# Supplementary material for: Trends in the use of the Internet for health purposes in Poland
Source: BMC Public Health. 2015 Feb 27;15:194. doi: 10.1186/s12889-015-1473-3 (PMC4349300; doi:10.1186/s12889-015-1473-3)

Figure S3 Profile of persons who consider the Internet as an important or not important source of medical information (SMI+/SMI-) in 2005 based on correspondence analysis

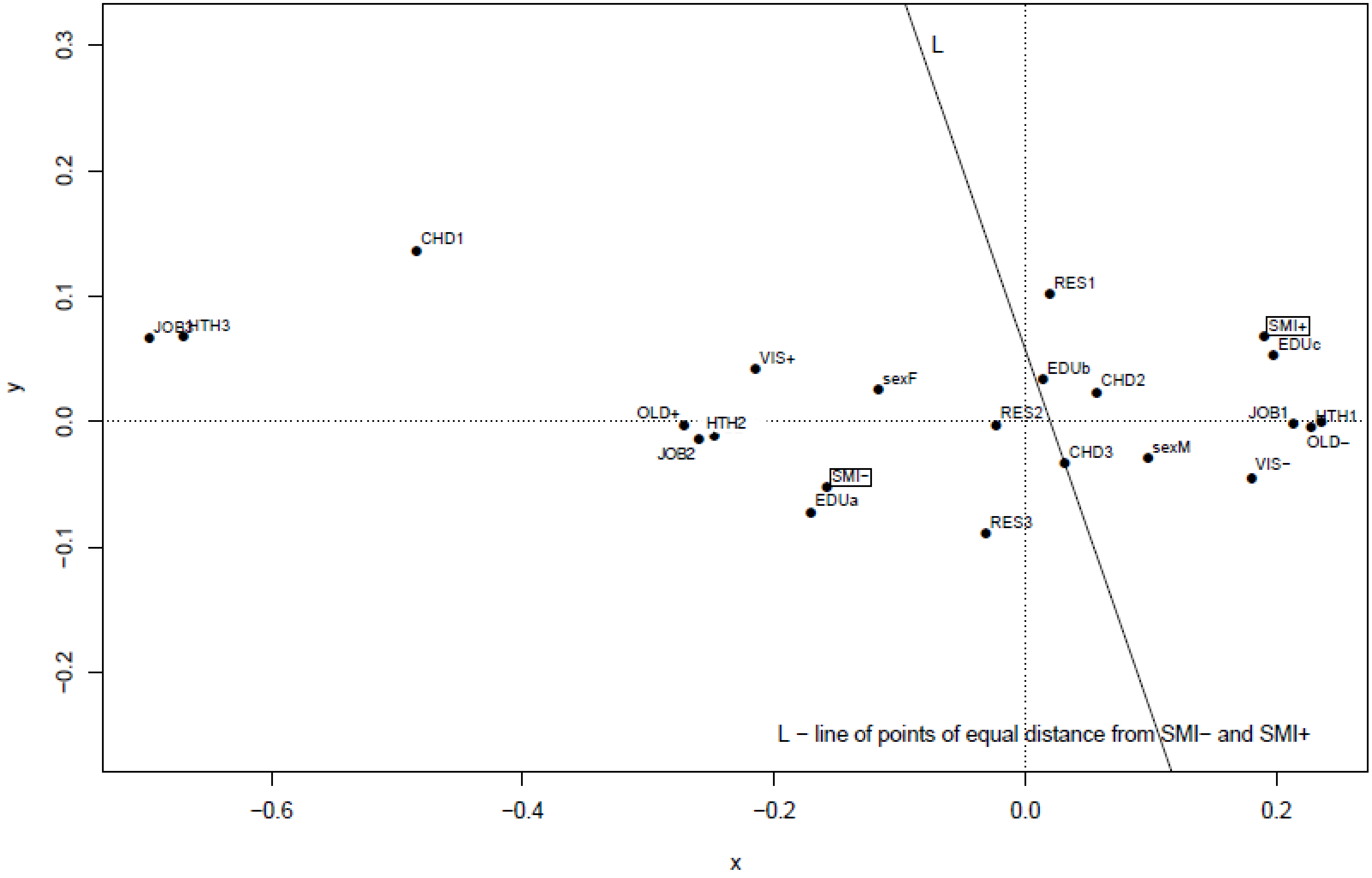

Figure S4 Profile of persons who consider the Internet as an important or not important source of medical information (SMI+/SMI-) in 2007 based on correspondence analysis

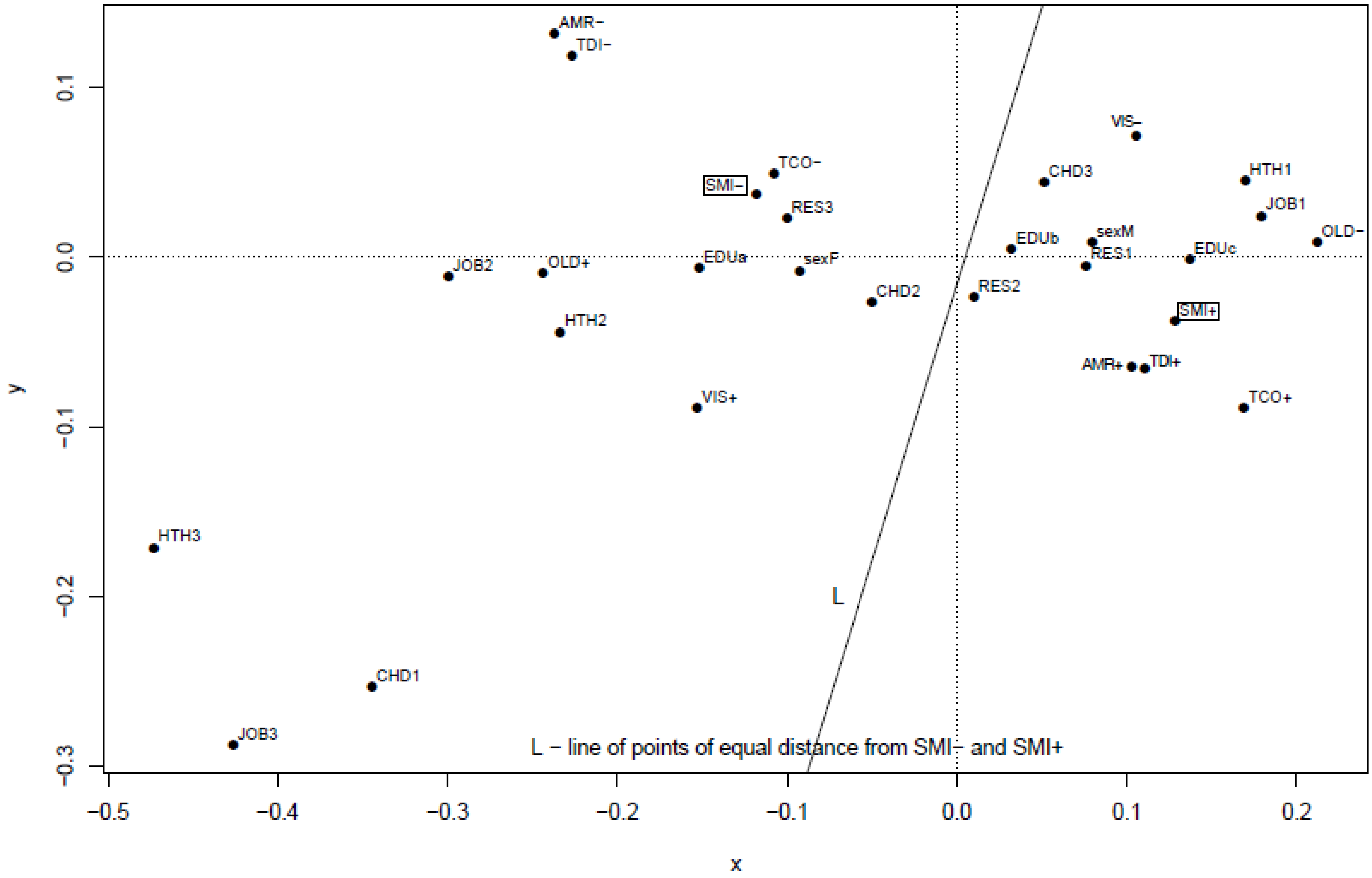

Supplement: Additional file 7: Figure S3. — Profile of persons who consider the Internet as an important or not important source of medical information (SMI+/SMI-) in year 2005 based on correspondence analysis. Figure S4. Profile of persons who consider the Internet as an important or not important source of medical information (SMI+/SMI-) in year 2007 based on correspondence analysis. [file 12889_2015_1473_MOESM7_ESM.pdf]
